# Increased Myocardial MAO-A, Atrogin-1, and IL-1β Expression in Transgenic Mice with Pancreatic Carcinoma—Benefit of MAO-A Inhibition for Cardiac Cachexia

**DOI:** 10.3390/biomedicines12092009

**Published:** 2024-09-03

**Authors:** Kira Stelter, Annalena Alabssi, Gabriel Alejandro Bonaterra, Hans Schwarzbach, Volker Fendrich, Emily P. Slater, Ralf Kinscherf, Wulf Hildebrandt

**Affiliations:** 1 Institute for Anatomy and Cell Biology, Department of Medical Cell Biology, Philipps-University of Marburg, Robert-Koch-Str. 8, 35032 Marburg, Germany; kiraschlick@gmail.com (K.S.); annalena.alabssi@outlook.de (A.A.); gabriel.bonaterra@staff.uni-marburg.de (G.A.B.); hans.schwarzbach@staff.uni-marburg.de (H.S.); ralf.kinscherf@staff.uni-marburg.de (R.K.); 2 Department of Visceral-, Thoracic- and Vascular Surgery, Philipps-University of Marburg, 35032 Marburg, Germany; vfendrich@schoen-klinik.de (V.F.); slater@staff.uni-marburg.de (E.P.S.)

**Keywords:** cancer cachexia, heart, KPC mouse, PDAC, capillary density, E3-ligases, harmine hydrochloride

## Abstract

Cancer cachexia (CC) continues to challenge clinicians by massively impairing patients’ prognosis, mobility, and quality of life through skeletal muscle wasting. CC also includes cardiac cachexia as characterized by atrophy, compromised metabolism, innervation and function of the myocardium through factors awaiting clarification for therapeutic targeting. Because monoamine oxidase-A (MAO-A) is a myocardial source of H_2_O_2_ and implicated in myofibrillar protein catabolism and heart failure, we presently studied myocardial MAO-A expression, inflammatory cells, and capillarization together with transcripts of pro-inflammatory, -angiogenic, -apoptotic, and -proteolytic signals (by qRT-PCR) in a 3x-transgenic (LSL-Kras^G12D/+^; LSL-TrP53^R172H/+^; Pdx1-Cre) mouse model of orthotopic pancreatic ductal adenoarcinoma (PDAC) compared to wild-type (WT) mice. Moreover, we evaluated the effect of MAO-A inhibition by application of harmine hydrochloride (HH, 8 weeks, i.p., no sham control) on PDAC-related myocardial alterations. Myocardial MAO-A protein content was significantly increased (1.69-fold) in PDAC compared to WT mice. PDAC was associated with an increased percentage of atrogin-1+ (*p* < 0.001), IL-1β+ (*p* < 0.01), COX2+ (*p* < 0.001), and CD68+ (*p* > 0.05) cells and enhanced transcripts of pro-inflammatory IL-1β (2.47-fold), COX2 (1.53-fold), TNF (1.87-fold), and SOCS3 (1.64-fold). Moreover, PDAC was associated with a reduction in capillary density (−17%, *p* < 0.05) and transcripts of KDR (0.46-fold) but not of VEGFA, Notch1, or Notch3. Importantly, HH treatment largely reversed the PDAC-related increases in atrogin-1+, IL-1β+, and TNF+ cell fraction as well as in COX2, IL-1β, TNF, and SOCS3 transcripts, whereas capillary density and KDR transcripts failed to improve. In mice with PDAC, increased myocardial pro-atrophic/-inflammatory signals are attributable to increased expression of MAO-A, because they are significantly improved with MAO-A inhibition as a potential novel therapeutic option. The PDAC-related loss in myocardial capillary density may be due to other mechanisms awaiting evaluation with consideration of cardiomyocyte size, cardiac function and physical activity.

## 1. Introduction

Cancer cachexia (CC) is experienced by a majority of cancer patients and by definition entails skeletal muscle wasting, which massively compromises the patients’ physical performance, pulmonary function, quality of life, tolerance to chemotherapy, and overall prognosis [1,2,3,4,5]. The still largely untreatable CC syndrome is highly prevalent in patients with gastrointestinal carcinoma, with the most rapid and devastating development observed with pancreatic ductal adenocarcinoma (PDAC) [1,5,6,7]. The phenotype of skeletal muscle wasting and its underlying pro-oxidative/-inflammatory and (neuro)endocrine mechanisms leading to net-proteolysis, apoptosis, and impaired regeneration via NF-κB-, STAT3-, myostatin-, insulin-Akt, or other pathways have been extensively studied within the last two decades [5,8,9,10,11,12,13,14,15,16]. However, relevant pro-cachectic triggers of myofibrillar catabolism may not spare the heart. Indeed, cardiac cachexia, i.e., decreased myocardial mass and function, has now been recognized as a largely understudied factor within CC [17,18] that independently impairs cancer patients’ prognosis [19], contributes to CC-related autonomic dysregulation [20], and is mutually interrelated to inactivity/skeletal muscle wasting [21]. In addition to myocardial atrophy and dysfunction, cardiac cachexia includes functional, structural, or metabolic remodeling, denervation, and mitochondrial dysfunction among other factors [18,22,23]. At present, the underlying mechanisms remain to be identified for effective therapeutic targeting [18] although they might not be fundamentally different from established factors driving skeletal muscle wasting [5,8,11].

The mitochondrial flavoenzyme monoamine oxidase-A (MAO-A) catalyzes the degradation of monoamine neurotransmitters and dietary amines, thereby generating H_2_O_2_, ammonium, and aldehyde [24,25,26]. Together with NADPH oxidases, xanthine oxidases, nitric oxide synthases, and the mitochondrial respiratory chain, MAO-A is an important source of ROS that is physiologically expressed in the myocardium [27,28,29,30]. High MAO-A expression or catecholamine-related activation and related oxidative stress have been strongly implicated in heart failure or experimental pressure overload, myocardial hypoxia-reoxygenation injury, diabetes-associated myocardial dysfunction, and cardiac aging [27,29,31,32,33]. For example, inhibition of MAO-A was shown to improve myocardial recovery from acute volume overload by aortal-caval fistula in rats [34] or from cardiac arrest [35] and to protect against catecholamine-induced arrhythmias [36].

Though largely understudied in the context of CC, MAO-A has been suggested to be a potential driver of and therapeutic target in skeletal muscle wasting. In an in-vitro model of steroid-induced skeletal muscle wasting, MAO-A was found to be massively upregulated [37]. Moreover, we have most recently shown that transgenic PDAC-bearing cachectic mice in comparison to WT mice revealed increased MAO-A protein expression in hindlimb skeletal muscle undergoing fiber atrophy [9,38]. Interestingly, selective reversible MAO-A inhibition with harmine hydrochloride (HH) significantly decreased pro-atrophic (atrogin-1, MuRF1) and pro-inflammatory (IL-1β, SOCS3) transcripts in hindlimb muscles of mice in this CC model. However, such desirable therapeutic effects failed to translate into reversal of muscle wasting, likely due to damage of neuromuscular junctions in fast-twitch skeletal muscle through the rather high HH dose of 30 mg kg^−1^ day^−1^ for 8 weeks [38].

Using this triple transgenic mouse model of PDAC-related CC, we presently aimed to study the role MAO-A in cardiac cachexia, hypothesizing that MAO-A inhibition by HH, i.e., lowering H_2_O_2_ generation, would attenuate pro-oxidative/-inflammatory/-atrophic alterations of the myocardium without the specific neuromuscular adverse effects observed in skeletal muscle [38].

High MAO-A expression/activity may also interfere conditionally and unpredictably with VEGF expression and angiogenesis, at least in cancer mouse models. MAO-A may contribute to HIF-1α stabilization, promoting VEGF expression, ROS production, tumorigenesis, and metastasis [39], whereas, under conditions of hypoxia and IL-6 receptor activation in breast cancer cells, enhanced VEGF expression is associated with MAO-A suppression [40]. Given the relevance of capillarization for cardiac function, not assessed so far with CC, we also determined myocardial capillary density with and without HH treatment in PDAC and WT mice.

The present study reports, for the first time, a significant increase in myocardial MAO-A expression with PDAC (-related cachexia) in combination with increases in the percentage of atrogin+-, IL-1β+-, and COX2+-cells as well as in related transcripts of IL-1β, COX2, TNF, and SOCS3 genes. As evidence for MAO-A as a causal pro-atrophic/-inflammatory driver and future therapeutic target, our data show that MAO-inhibition by HH mostly reversed almost all of these pro-cachectic/-inflammatory impairments.

## 2. Material and Methods

### 2.1. Transgenic Mouse Model of PDAC

The presently studied triple transgenic mouse model of PDAC—introduced and provided by Hingorani et al.—is based on the clinically highly PDAC-relevant point mutations of transformation-related protein 53 (Trp53, R172H/+: arginine/histidine substitution in codon 172) and Kirsten rat sarcoma (Kras, G12D/+: glycine/aspartate substitution in codon 12) [41,42] and develops muscle wasting as described [9,38]. Activation of the combined mutants upon crossbreeding with the pancreas specific Pdx-1-Cre promoter leads to orthotopic PDAC development via progressive inflammation-driven precancerous lesions (pancreatic intraepithelial neoplasia, PanIN grades 1–3). WT mice with a BL/6 background served as controls. All mice were maintained by the Biomedical Research Centre of the University of Marburg under conditions of food and water supply ad libitum as well as a periodic day–night cycle of 12 h.

Out of 42 mice in total (23 WT and 19 PDAC mice), subgroups of 11 WT and 9 PDAC mice were treated with HH (30 mg/kg/day, i.p.) for two months, starting at the age of three months. After the treatment, mice were sacrificed, their hearts removed, and non-apical/-basal samples of the left myocardium for qT-PCR or Western blotting as well as transversal sections at maximal circumference for cryo-sections (embedded in Tissue-Tek™, Sakura Finetek, Stauffen, Germany, and submersed in precooled isopentane) were snap-frozen using liquid nitrogen. The histopathological diagnosis of PDAC or normal pancreas was independently assessed by two experienced investigators. The study was approved by the Regional Commission Giessen (MR 20/11-Nr.70/2009) and conducted according to the regulations for animal experiments at the Philipps University, Marburg.

### 2.2. Western Blotting of MAO-A Expression

Ventricular left myocardial samples were lysed in radioimmunoprecipitation assay (RIPA) buffer at pH 7.5 (Cell Signaling Technology Europa, Leiden, The Netherlands) with protease/phosphatase inhibitors (Cell Signaling Technology, Boston, MA, USA) and their total protein concentrations determined by means of the Pierce BCA (bicinchoninic acid) assay (Thermo Scientific, Rockford, IL, USA) according to the manufacturer’s instructions. Proteins were loaded onto pre-cast polyacrylamide NuPAGE^®^ 4–12% Bis-Tris gels (Life Technologies GmbH, Darmstadt, Germany). Following SDS-PAGE, proteins were transferred onto 0.45 µm nitrocellulose membranes (Millipore; Billerica, MA, USA). Membranes were stained for MAO-A (rabbit monoclonal anti-MAO-A antibody, ab126751, Abcam, Cambridge, UK) and for α-tubulin (1:2000, ab4074, Abcam, Cambridge, UK) by incubation with the respective immunoreactive primary antibodies overnight at 4 °C in blocking buffer. After washing with TBS 0.1% Tween 20, membranes were incubated with ECL anti-rabbit IgG, horseradish peroxidase (HRP) secondary antibody (MA9340, GE Healthcare, Amersham, UK). The peroxidase reaction was visualized with ImmobilonTM Western (HRP) substrate (Merck Chemicals GmbH. Darmstadt, Germany). The intensity of WB bands was quantified using ImageJ/Fiji software (version 1.54f) from the National Institutes of Health (Bethesda, MD, USA).

### 2.3. Immunolocalization of Atrogin-1+, IL-1β+, TNF+, COX2+, and CD68+ Cells

Transversal or longitudinal cryo-sections of the ventricular left myocardium were fixed by 5% paraformaldehyde (PFA) for 10 min, blocked with 1% normal swine serum (NSS) at 37 °C, and incubated with primary rabbit anti-mouse polyclonal antibodies against atrogin-1 (FBXO32, MAFbx, 1:200, ab74023, Abcam, Cambridge, UK), against IL-1β (1:50, ab9722, Abcam, Cambridge, UK), TNF (1:100, ab6671, Abcam, Cambridge, UK) or against COX2 (1:200, ab15191, Abcam, Cambridge, UK), and primary rat anti-mouse primary monoclonal antibodies against CD68 (1:50, MCA1957, AbD Serotec, Kidlington, UK). Their respective detection was enabled by anti-rabbit IgG (1:200, ZRH1158, Linaris, Biological Products, Dossenheim, Germany) or anti-rat IgG (1:200, STAR72, AbD Serotec, Kidlington, UK) secondary antibodies that were conjugated with horseradish peroxidase (HRP) for catalyzation of oxidation with H_2_O_2_ of 3,3′-diaminobenzidine (DAB) as a chromogenic substrate. Nuclei were counterstained with Mayer’s hematoxylin. Using digital images (200×) obtained by the Zeiss Axio Imager M2 microscope combined with Axio-Cam HRc/AxioVision, the total number of immunohistochemically positive cell and counterstained nuclei were analyzed in three rectangular areas (0.145 mm^2^) of both transversal (*n* ≥ 3) and longitudinal (*n* ≥ 3, except for atrogin-1 staining) representative tissue sections using ImageJ processing package Fiji software 1.54f. The density of immunolocalized cells was assessed in terms of percentage (%) of counterstained hematoxylin+ nuclei in total (to enable control for possible changes in cardiomyocyte size).

### 2.4. Myocardial Capillary Density

Ventricular left myocardial capillary density (CD) was determined by histochemical visualization of capillaries in 7-µm cryo-cross-sections fixed in 4% paraformaldehyde/phosphate-buffered saline (PFA/PBS) for 10 min at room temperature as previously described [9,38]. Briefly, after blocking endogenous peroxidases with hydrogen peroxide (0.05% in PBS; pH 7.4), the cross-sections were incubated for 30 min at 37 °C with 40 μg/mL horseradish peroxidase-conjugated Isolectin B4 of Bandeiraea simplicifolia (BSI-B4, Sigma-Aldrich Co. LLC, St. Louis, MO, USA) in PBS or with D-galactose for negative control staining, and 3-3′-diaminobenzidine (DAB, Merck-Sigma-Aldrich Co. LLC, St. Louis, MO, USA) was used for detection of capillaries. Nuclei were counterstained with Mayer’s hematoxylin (Carl Roth GmbH & Co. KG, Karlsruhe, Germany). Digital images (200×) were obtained by the Zeiss Axio Imager M2 microscope (Carl Zeiss AG; Oberkochen, Germany) combined with Axio-Cam HRc/AxioVision software Release 4.8.2 (Carl Zeiss GmbH, Oberkochen, Germany). Three separate rectangular cross-sectional areas (0.145 mm^2^) were used to assess CD in terms of counts of capillaries (diameter ≤ 7.5 µm) per area using ImageJ/Fiji software (National Institute of Health, Bethesda, MD, USA).

### 2.5. Quantitative Reverse Transcription Polymerase Chain Reaction (qRT-PCR)

RNA extraction was carried out using peqGOLDTriFast™ (VWR International GmbH, Darmstadt, Germany) according to the manufacturer’s instructions. RNA concentration was measured via optical density (OD, 260 nm) as well as its purity assessed (OD260 nm/OD280 nm, with a ratio of 1.8 to 2.0 accepted as pure) using a NanoDrop 2000c spectrophotometer (Thermo Fisher Scientific Inc., Waltham, MA, USA). A high RNA integrity (RIN, between 8 and 10) for reverse transcription (RT) was confirmed by means of an RNA 6000 NanoChip kit on an Agilent 2100 Bioanalyzer (Agilent Technologies Inc., Santa Clara, CA, USA). An aliquot of total RNA was treated with one unit DNAse (Thermo Fisher Scientific Inc.) for 30 min at 37 °C and RT performed for 1 h at 42 °C using oligo primer (dT)_12–18_ (Agilent Technologies Inc.), 20 units of the reverse transcriptase included in the Affinity Script multiple-temperature cDNA synthesis kit (Agilent Technologies Inc.), 24 units of Ribo Lock™ RNAse inhibitor (Thermo Fisher Scientific Inc.), and 4 mM dNTP mix (Agilent Technologies Inc.). The cDNA was used for qRT-PCR using the QuantiTect-primer assays (Table 1) (Qiagen N.V., Venlo, Netherlands) combined with Takyon™ Low Rox Probe Master-Mix dTTP Blue (Eurogentec, Seraing, Belgium) or Agilent™ Brilliant III Ultra-Fast SYBR^®^ Green QPCR Master-Mix (Agilent Technologies Inc.). The thermal profile consisted of 3 min at 95 °C followed by 45 cycles at 95 °C for 10 s and 60 °C for 20 s. The qPCR and data analyses were performed using the Stratagene Mx3005P™ qPCR System (Agilent Technologies, Inc.). The relative amount within each sample was calculated by linear regression analysis from their respective standard curves generated from a cDNA pool. The specificity of the amplified product was confirmed by the melting curve analysis (55–95 °C). The myocardial expression of RPLP0 was selected out of potential candidate genes, including β-actin, GAPDH, etc., as a housekeeping gene using Normfinder software version 20 as described [38] (Table 1).

### 2.6. Statistical Analyses

Data are presented as mean ± standard error of the mean (SEM) for the groups WT, WT-HH, CA, and CA-HH. To detect a significant and independent impact of PDAC and/or of HH treatment, a two-factorial ANOVA (with or without age or sex as covariates) was applied to the total study population and *p*-values presented for the effect of PDAC (CA and CA-HH group) vs. WT (WT and WT-HH groups) and/or for the effect of HH (WT-HH and CA-HH group) vs. untreated mice (WT and CA group) as well as for the interaction of both these factors in tables and figure legends. Significant differences between two groups were assessed (posthoc) by the Student’s t-test or, in case of no normal distribution, by the Mann–Whitney U test, and indicated within the graph by the symbol * (for PDAC effects) or the symbol # (for HH effects). A *p* < 0.05 was considered to be statistically significant. Given the explorative (hypothesis-generating) nature of the study, no Bonferroni correction was applied. All statistical procedures were performed by SPSS (version 27, IBM Munich, Munich, Germany).

## 3. Results

### 3.1. Group Characteristics

As presented in Table 2, the CA group (with histopathological confirmation of PDAC) showed no significant weight loss as compared to the WT group (control with normal pancreatic histology). Obviously, the significant PDAC-related skeletal muscle wasting, as recently reported in this mouse model [9,38], was not reflected by a significant weight loss, likely due to variable tumor, metastasis or fat mass, extent of ascites, or other factors. PDAC-related weight loss did tend to be higher in the male compared to the female CA group as recently reported [9]. This is in line with their larger muscle mass in terms of percentage of body weight. Overall, HH treatment had no significant impact on body weight; however, the CA-HH group revealed significantly lower values by 2.4 g. Age did not significantly impact body weight, although the CA group was significantly older than the WT and CA-HH groups by ca. 7 and 4 weeks, respectively, while the WT-HH group was older than the WT group by ca. 2 weeks (Table 2).

### 3.2. Increased MAO-A Protein Content in PDAC Compared to WT Mice

**Figure 1:**
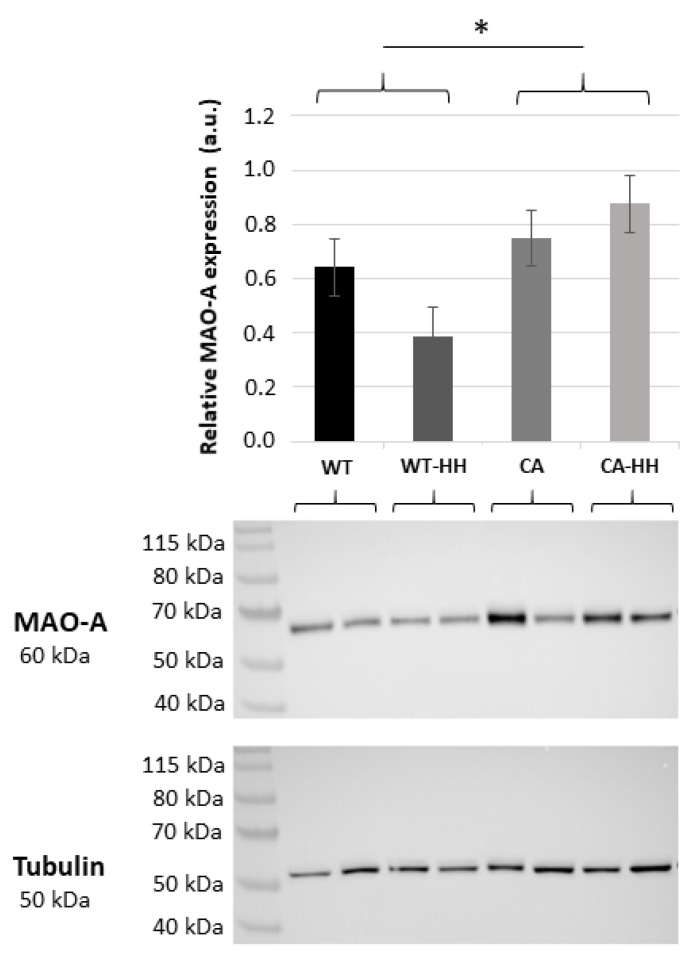
Relative MAO-A protein content in the ventricular left myocardium of untreated or HH-treated wild type (WT, WT-HH) and PDAC-bearing mice (CA, CA-HH). Data are given as mean ± SEM. Two-factorial ANOVA detected a significant increasing effect of factor CA (*p* = 0.02) but no impact of factor HH (*p* = 0.61) on MAO-A content; (*n* = 4 independent experiments); * for *p* < 0.05 CA + CA-HH vs. WT + WT-HH. Representative Western blots for MAO-A and for tubulin (loading control) are given below with indications of the sample group assignments.

Relative MAO-A protein content in the left myocardium (Figure 1) showed a variable, however, overall significant 1.69-fold increase in PDAC-bearing (CA and CA-HH) as compared to WT (WT and WT-HH) mice. ANOVA confirmed a significant effect of PDAC independent of the factors HH treatment, age, or gender, all of which were without significant impact compared to MAO-A protein expression.

This PDAC-related increase in protein level was significant, with normalization for both tubulin (Figure 1) and Ponceau staining of total protein abundance. Notably, no parallel PDAC-related increase in myocardial MAO-A (or MAO-B) transcripts was observed (Table 3).

### 3.3. Percentage of Atrogin-1+ Cells Is Increased in PDAC and Reversed by HH-Treatment

**Figure 2:**
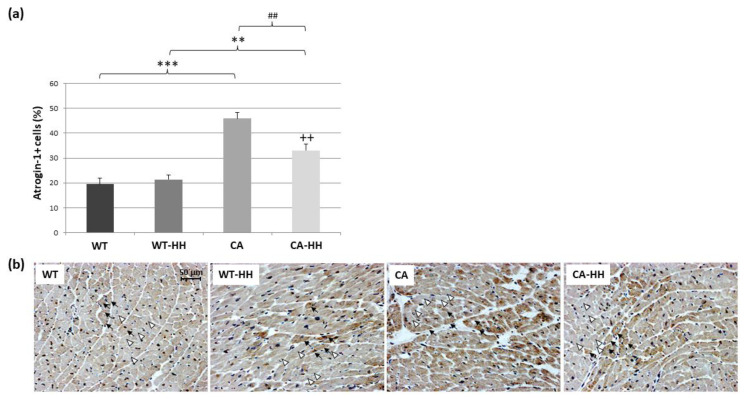
Atrogin-1+ cells in the ventricular left myocardium of WT (*n* = 7), WT-HH (*n* = 11), CA (*n* = 7), and CA-HH (*n* = 9) mice. (**a**) Percentage of atrogin-1+ cells. Data are given as mean ± SEM. Two-factorial ANOVA detected a significant opposing impact of factor CA (*p* < 0.001) and factor HH treatment (*p* < 0.05), with significant interaction (*p* < 0.01) within the total study population. ** *p* < 0.01, *** *p* < 0.001 CA vs. WT or CA-HH vs. WT-HH; ## *p* < 0.01, WT-HH vs. WT or CA-HH vs. CA; ++ *p* < 0.01 CA-HH vs. WT. (**b**) Representative photos of left myocardial cross-sections of WT, WT-HH, CA, and CA-HH mice indicating five examples of atrogin-1+ (black arrows) and atrogin-1-negative cardiomyocytes (empty arrow heads).

The density of atrogin-1+ or other immunolocalized (see below) cells was expressed in terms of percentage of total hematoxylin+ nuclei, which, notably, did not significantly differ in terms of count per area (n/mm^2^) between the four groups of WT (2037 ± 74), WT-HH (2013 ± 54), CA (1861 ± 109), and CA-HH (2113 ± 77). As a main finding (Figure 2a,b), there was a highly significant more than two-fold PDAC-related increase in percentage of atrogin-1+ cells (atrogin-1+ cells in % of hematoxylin+ cells) in myocardial cross-sections. Immunohistochemical staining shows that the PDAC-related increase in atrogin-1 expression actually occurs within cardiomyocytes (Figure 2b). HH treatment significantly but incompletely reversed the increase in percentage of atrogin-1+ cardiomyocytes to a level that remained significantly above that of the WT mice (control).

In WT mice, HH treatment appeared to have little effect on percentage of atrogin-1+ cardiomyocytes. ANOVA confirmed significant, independent opposing effects of PDAC and of HH, while age and sex had no significant impact. Notably, there was a significant positive correlation between percentages of atrogin-1+ cardiomyocytes (r = 0.687 *p* = 0.001) and MAO-A protein contents among the total study population, i.e., irrespective of the (incompletely reversing) effect of HH. In contrast, myocardial atrogin-1 transcript levels were found to be largely unaffected by PDAC or HH treatment in pooled analyses (Table 3).

Moreover, the pooled transcript levels of MuRF1, another relevant pro-atrophic ubiquitin E3-ligase, were unaffected by PDAC as well but tended to be higher with HH treatment in the WT and the CA group (Table 3).

Regarding apoptosis as another factor of cardiac cachexia, transcripts of Casp3 and BCL-2 were largely unaffected by PDAC and HH treatment except for a <0.50-fold decrease with HH treatment in the CA-HH group compared to the CA or the WT group (Table 3).

### 3.4. Upregulation of IL-1β, TNF, COX2, CD68, and SOCS with PDAC and Differential Anti-Inflammatory Effect of HH Treatment

**Figure 3:**
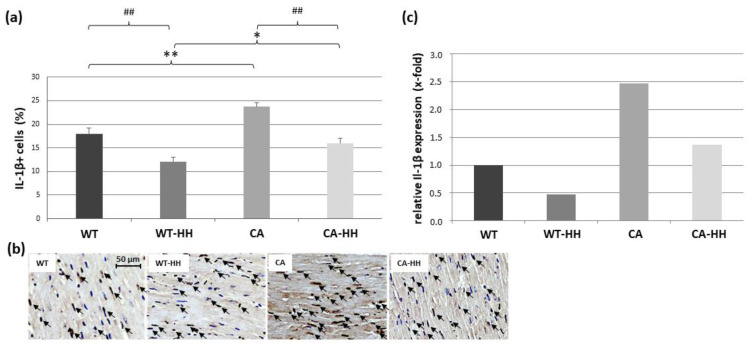
ILβ+ cells in the left ventricular myocardium of WT, WT-HH, CA, and CA-HH mice. (**a**) Percentage of IL-1β+ cells. Data are given as mean ± SEM. Two-factorial ANOVA detected significant opposing impacts of factor CA (*p* < 0.001) and factor HH treatment (*p* < 0.001) without significant interaction within the total study population. * *p* < 0.05, ** *p* < 0.01 CA vs. WT or CA-HH vs. WT-HH; ## *p* < 0.01, WT-HH vs. WT or CA-HH vs. CA. (**b**) Representative photos of left myocardial cross-sections of WT, WT-HH, CA, and CA-HH mice showing IL-1β+ cells (arrows). (**c**) x-fold expression of IL-1β transcripts in the left myocardium of WT-HH, CA, and CA-HH mice relative to WT mice as assessed in pooled samples.

Since IL-1β is a well-established pro-inflammatory trigger of muscle wasting, it was presently considered as another main outcome variable in this context of cardiac cachexia. As presented in Figure 3a,b, percentage of IL-1β+ cells (TNF+ cells in % of hematoxylin cells) significantly increased in PDAC overall, as reflected in CA or CA-HH mice compared to WT or WT-HH mice, respectively. Moreover, this PDAC-related increase in percentage of IL-1β+ cells was significantly reduced with HH-treatment overall and this effect was detectable in comparison to both WT and CA mice, as well. These significantly opposing impacts, PDAC and HH treatment, showed no significant interaction according to ANOVA and were independent of age or sex. In line with these data, IL-1β transcript levels (Figure 3c, Table 3) were found to be almost 2.5-fold higher in CA than in WT mice and even more pronounced in CA-HH than in WT-HH mice, while HH treatment considerably decreased these levels in both PDAC-bearing and WT mice.

**Figure 4:**
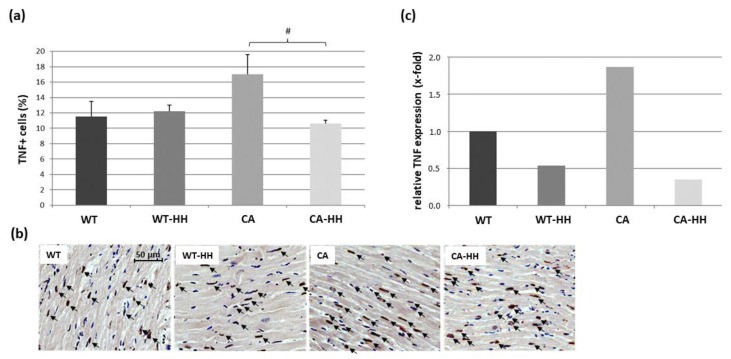
TNF+ cells in the ventricular left myocardium of WT, WT-HH, CA, and CA-HH mice. (**a**) Percentage TNF+ cells. Data are given as mean ± SEM. Two-factorial ANOVA applied to the total study population detected no significant impact of factor CA or factor HH treatment; however, revealed a significant interaction (*p* < 0.05) between these factors, as reflected by a significant decrease in increased TNF+ cell density through HH in PDAC-bearing mice. # *p* < 0.05 (posthoc) CA-HH vs. CA. (**b**) Representative photos of left myocardial cross-sections of WT, WT-HH, CA, and CA-HH mice showing TNF+ cells (arrows). (**c**) x-fold expression of TNF transcripts in the left myocardium of WT-HH, CA, and CA-HH relative to WT mice as assessed in pooled samples.

As seen in Figure 4a,b, the percentage of TNF+ cells showed a, by trend, 1.45-fold increase in the CA compared to the WT group, which was counteracted by HH treatment, leading to a significant decrease in PDAC-bearing mice. These effects were confirmed in terms of a significant interaction of the factors PDAC and HH treatment according to ANOVA. In line with these data, TNF transcript levels showed a 1.87-fold increase in the CA group vs. the WT group and a strong opposing effect through HH treatment, which decreased TNF transcripts to largely below WT group, i.e., control level (Figure 4c, Table 3). The percentage of TNF+ cells were found to be unrelated to MAO-A protein expression.

**Figure 5:**
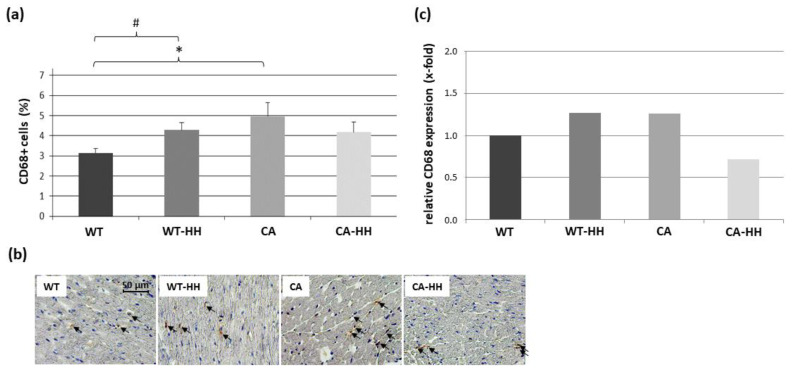
CD68+ cells in the left ventricular myocardium of WT, WT-HH, CA, and CA-HH mice. (**a**) Percentage CD68+ cells. Data are given as mean ± SEM. Two-factorial ANOVA detected no significant impact of factor CA or factor HH treatment; however, revealed a significant interaction (*p* ≤ 0.05) between these factors within the total study population. * *p* < 0.05 CA vs. WT. # *p* < 0.05 WT-HH vs. WT. (**b**) Representative photos of left myocardial cross-sections of WT, WT-HH, CA, and CA-HH mice showing CD68+ cells (arrows). (**c**) X-fold expression of CD68 transcripts in WT-HH, CA, and CA-HH relative to WT mice as assessed in pooled samples.

As presented in Figure 5a,b, moderate but significant PDAC- and HH-related increases in the percentage of CD68+ cells were detected in untreated mice, which was reflected by a significant interaction but no significant impact of factor CA and factor HH within the total study population. The CD68 transcript levels (Figure 5c, Table 3) revealed a similar trend but no relevant change through PDAC or HH treatment.

**Figure 6:**
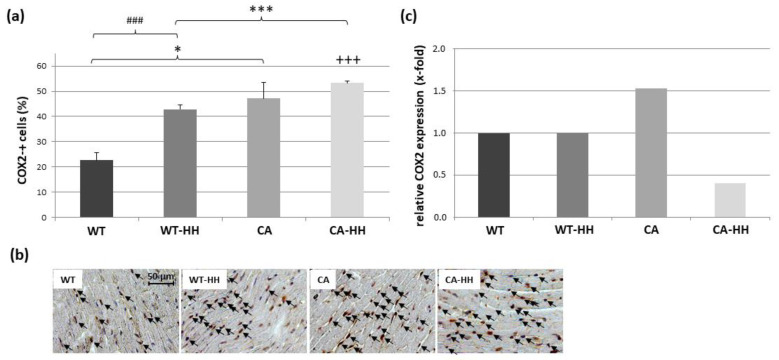
COX2+ cells in the left ventricular myocardium of WT, WT-HH, CA, and CA-HH mice. (**a**) Percentage of COX2+ cells. Data are given as mean ± SEM. Two-factorial ANOVA detected significant impacts of factor CA (*p* < 0.001) and factor HH treatment (*p* < 0.001) with significant interaction (*p* < 0.05) within the total study population. * *p* < 0.05, *** *p* < 0.001 CA vs. WT or CA-HH vs. WT-HH; ### *p* < 0.001 WT-HH vs. WT or CA-HH vs. CA. +++ *p* < 0.001 CA-HH vs. WT. (**b**) Representative photos of left myocardial cross-sections of WT, WT-HH, CA, and CA-HH mice showing COX2+ cells (arrows). (**c**) X-fold expression of COX2 transcripts in the left myocardium of WT-HH, CA, and CA-HH mice relative to WT mice as assessed in pooled samples.

Figure 6a,b shows a significant increase in the percentage of COX2+ cells with PDAC by ANOVA, which was detectable in the CA and CA-HH groups as compared to the WT and WT-HH groups, respectively. Overall, HH treatment resulted in a significant additional increase in the percentage of COX2+ cells whereby a significant interaction between the factors CA and HH indicated a positive synergism of both effects. The effect of HH treatment on percentage COX2+ cells, however, reached significance in WT but not in CA mice. On the level of transcripts (Figure 6c, Table 3), COX2 showed a 1.53-fold upregulation in the CA group compared to the WT group, which was massively reversed by 0.27-fold through HH treatment. No such effect was observed in the WT-HH compared to the WT group.

As shown in Table 3, the transcript level of SOCS3, also implicated in pro-cachectic signaling, was found to be 1.64-fold upregulated in the CA group compared to the WT group, whereas HH treatment decreased these <0.5-fold below WT group (control) level.

### 3.5. Effect of PDAC and HH on Myocardial Capillary Density and Angiogenic Signals

**Figure 7:**
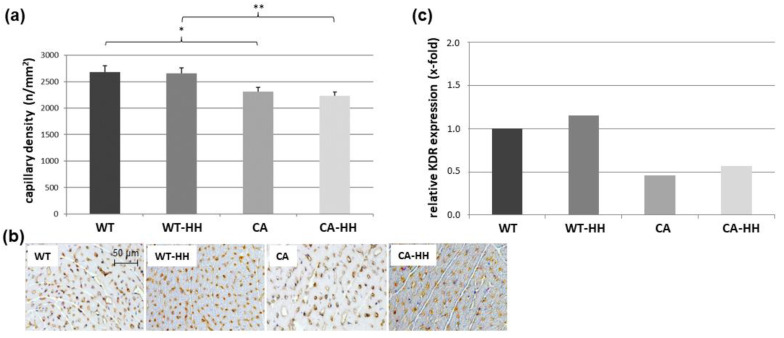
Capillary density in the ventricular left myocardium of WT, WT-HH, CA, and CA-HH mice. (**a**) Density of capillaries (n/mm^2^, diameter ≤ 7 µm, stained for α-lectin). Data are given as mean ± SEM. Two-factorial ANOVA detected a significant impact of factor PDAC (*p* < 0.001). but not factor HH. * *p* < 0.05, ** *p* < 0.01 CA vs. WT or CA-HH vs. WT-HH. (**b**) Representative photos of left myocardial cross-sections of WT, WT-HH, CA, and CA-HH mice showing α-lectin+ capillaries. (**c**) X-fold expression of KDR transcripts in the left myocardium of WT-HH, CA, and CA-HH relative to WT mice as assessed in pooled samples.

As shown in Figure 7a,b, overall PDAC was associated with a highly significant reduction in capillary density (−15%), which was detectable between the CA and WT groups (−14%) as well as between the CA-HH and WT-HH groups (−16%). In contrast, HH treatment yielded no significant changes in capillary density. Regarding transcript levels of relevant angiogenic signals, VEGFA remained largely unaffected by PDAC or HH treatment (Table 3). However, a 0.48-fold downregulation of its receptor KDR was observed in the CA group compared to the WT group (Figure 7c, Table 3). HH treatment had little effect on KDR in PDAC-bearing or WT mice.

The transcripts of Notch1 and Notch3 displayed a trend toward PDAC-related downregulation, which was somewhat reinforced by HH treatment (Table 3).

## 4. Discussion

Cancer cachexia involves catabolic, mostly inflammatory mediators, that not only trigger skeletal muscle wasting but may also promote cardiac cachexia. This includes myocardial atrophy, dysfunction, and related remodeling that critically compromises cancer patients’ prognosis and quality of life [17,18,19]. The present study aimed to evaluate the role of MAO-A, a novel potential therapeutic target in CC, in the context of cardiac cachexia [38].

Using an established triple transgenic mouse model with orthotopic PDAC [41,42] compared to WT mice, we demonstrate for the first time that PDAC (-related CC) is associated with a significantly higher protein expression of MAO-A in the left myocardium. As a mitochondrial source of H_2_O_2_, MAO-A (like MAO-B) contributes to oxidative stress in the myocardium as well as in skeletal muscle [27,28,29,30,33,37,43]. High MAO-A expression or activity is clearly implicated in several cardiac conditions [27,29,31,32] that benefit from MAO-A inhibition [34,35,36]. As yet, MAO-A has not been addressed in cardiac cachexia; however, we recently reported, in the presently studied PDAC-related mouse model of CC, that MAO-A protein expression was significantly increased in skeletal (gastrocnemius) muscle. Furthermore, MAO-A inhibition with HH significantly attenuated transcripts of pro-inflammatory IL-1β in the gastrocnemius muscle and of the pro-atrophic E3-ligases atrogin-1 and MuRF1 in the soleus muscle without changing MAO-A protein expression levels [38]. Unfortunately, this potentially anti-cachectic effect of HH failed to translate into a reversal of muscle skeletal wasting, likely due to a compromised neuromuscular junction as an adverse effect of HH. Given the principle difference in cardiac innervation, we presently hypothesized that HH treatment could offer a therapeutic option by attenuating possible CC-related upregulation of E3 ligases and upstream cytokine triggers responsible for myofibrillar breakdown in myocardium as well as skeletal muscle [17,44].

As a main finding, we observed a PDAC-related highly significant increase in myocardial atrogin-1+ cell fraction (%) that was significantly, though incompletely, reversed by the reversible selective MAO-A inhibitor HH. Notably, these opposing effects of PDAC and HH treatment were also well reflected in terms of atrogin-1+ cell density (n/mm^2^). However, this may be more biased through possible cardiomyocyte atrophy (not assessed) than with normalization to the total count of nuclei, which was found to be similar among the four groups. Notably, atrogin-1 (and MuRF1) transcript levels were largely unaffected by PDAC or HH treatment, potentially indicating feedback regulation at high atrogin-1 protein abundance. Nevertheless, these data suggest that enhanced H_2_O_2_ production through increased MAO-A expression or (e.g., catecholamine-induced) activity may well contribute to upregulation of pro-atrophic atrogin-1 in cardiomyocytes of PDAC-bearing mice, since MAO inhibition mostly reversed atrogin-1+ expression, which was immunolocalized within cardiomyocytes. In support of such a potentially causal role for MAO-A in atrogin-1 upregulation, there was the finding of a significant and positive relationship between percentage atrogin-1+ cells (r = 0.687 *p* = 0.001) and MAO-A protein expression, irrespective of HH treatment. Notably, HH did not affect MAO-A protein or RNA expression but was presently assumed to reduce atrogin-1+ cells via inhibition of MAO-A activity (not measured).

Atrogin-1 is a well-established pro-atrophic [16,17,44] or anti-hypertrophic [45,46] myocardial ubiquitin E3 ligase that is under converging control of the myostatin-ActRIIB-Smad 2/3-, IGF-1-Akt-, or NF-κB-dependent FoxO3 transcription factor [8,12,13,47,48], which clearly regulates the cardiomyocyte size [49]. For example, increased RNA and protein expression of atrogin-1 together with MuRF-1 were found with myocardial or skeletal muscle wasting in a Lewis lung cancer mouse model and attributable to NF-κB activation due to increased Iκκβ and phospho-p65 expression [17], as NF-κB inhibition via luteolin largely reversed E3 ligases and losses in cardiac and skeletal muscle mass. In the present study, NF-κB signaling in response to H_2_O_2_ may well convey the observed atrogin-1 upregulation [50,51] since it can be inhibited together with cytokine expression by HH [52], as presently supported by the reversal of upregulated IL-1β and TNF. These cytokines indeed trigger E3 ligase upregulation and, in turn, reinforce their transcription via NF-κB activation [5,8,12,15,51,53,54,55,56]. Systemic or local upregulation of TNF may have triggered oxidative stress and further cytokine release, all of which can be attenuated by antioxidants [57] and likely by inhibition of MAO-A activity, which notably itself responds to oxidative stress [44]. In addition to NF-κB, the complex downstream effects of PDAC may also involve inhibition of the IRS-AK pathway by TNF or Smad 2, 3 or other factors [8,12,58]. Notably HH treatment also led to marked reversal of the upregulated SOCS3 transcripts to below WT (control) levels. SOCS3 expression can occur in response to cytokine-triggered STAT3 signaling or H_2_O_2_ and likely contributes to proteasomal degradation [9,59,60,61] and cardiac remodeling, e.g., after myocardial infarction [62]. Although interconnected, all pro-cachectic pathways involved appear to finally converge in atrogin-1 expression, which presently was effectively reduced by HH treatment. Thereby, atrogin-1 may co-activate its own transcription factor via Akt, what can be considered to be a positive feedback mechanism [45]. Notably, however, an overly simplistic total inhibition of H_2_O_2_ may not be desirable with all signaling steps, as certain prolonged high levels of H_2_O_2_ may promote cardiac hypertrophy rather than atrophy [63] and the anabolic IGF-1-Akt pathway may be activated by H_2_O_2_, at least in myotubes [64].

Regarding the observed (2.08-fold increase) increased fraction of COX2-expressing cells with PDAC, in accordance with transcript levels, one may assume that a PDAC-related infiltration (1.57-fold increase) of CD68+ cells (macrophages) into the myocardium occurs, which may locally trigger COX2 expression in various cells including cardiomyocytes. Why the HH-induced reduction of COX2-transcript levels does not translate into reduced COX2-protein expression remains unclear at present. Notably, COX2 upregulation may have a time-dependent cardioprotective role under conditions of H_2_O_2_ treatment [65] and may be required for hypertrophy, at least in skeletal muscle [66].

To our knowledge, myocardial capillary rarefication has not been previously reported in the context of cardiac cachexia but may be highly relevant for cardiac dysfunction. The significant decrease in capillary density by −17% may even underestimate the actual loss in capillary contacts per fiber, as any cardiomyocyte atrophy will enhance, per se, capillary density. The factors behind PDAC-related capillary losses despite stable VEGFA expression presently remains elusive. However, they may include the observed reduction in KDR that, like capillary density, failed to improve with HH treatment. A high PDAC-related TNF expression may, in theory, convey an anti-angiogenic effect [67]; however, this should involve lower VEGFA expression levels, which were presently not observed. Moreover, the HH-related decreases in TNF were presently not associated with improved capillarization. However, as a note of caution, any anti-atrophy effect on fiber size via HH treatment would result in a decreased capillary density, i.e., mask any potential neo-angiogenesis. As COX2 plays a role in myocardial angiogenesis, its regulation with PDAC and HH (downregulated on the RNA level) may warrant further studies including the function of inflammatory macrophages [68,69].

In addition to MAO-A, HH treatment may also inhibit the x-chromosomal multifunctional dual-specificity tyrosine phosphorylation-regulated kinases 1A (DYRK1A) and 1B (DYRK1B /MIRK) [70]. Like MAO-A, DYRK1A and 1B may be overexpressed in heart failure and impair hyperplasia and mitochondrial function, respectively [71,72]. However, in contrast to MAO-A, DYRK1B may have an antioxidative role promoting myogenesis and autophagy, at least in skeletal muscle [73,74]. Thus, HH inhibition of MAO-A and DYRK1B may result in differential and not thoroughly beneficial effects. This may also be true for DYRK1A, which promotes an angiogenic response by interfering with VEGF-dependent activation of the Ca^2+^/nuclear factor of activated T-cells (NFATs) pathway in endothelial cells [75]. DYRK1A inhibition may thus be anti-angiogenic and oppose any potential pro-angiogenic effect of MAO-A inhibition [40], in line with the failure of HH to improve capillarization. However, DYRK1A inhibition may facilitate cardiac repair and function by increasing cardiomyocyte cycling/hyperplasia following ischemia–reperfusion stress [71]. Nevertheless, capillary rarefication may generally arise from cardiac detraining/inactivity as a result of both CC-related and the recently reported HH-related skeletal muscle atrophy [38].

As a limitation, the presently studied HH intervention in PDAC and WT mice was not sham-controlled by a daily i.p. application of the HH medium, which by itself might have had an effect. However, the selective reversal of PDAC-related increases in atrogin-1, TNF, or CD68 (as reflected by the significant interaction of PDAC and HH in ANOVA), supported the hypothesis that inhibition of the overexpressed MAO-A may be the main factor behind such beneficial changes. Together with further limitations like large variability in MAO-A expression, pooled PCR analyses, some gender imbalance between the groups, and the limited sample size associated with the risks of type 1 and type 2 errors, our present data may be considered hypothesis-generating. In view of lacking therapeutic options of CC, the present data call for larger controlled studies with consideration of gender, systemic inflammation, physical activity, cardiomyocyte size, myocardial muscle mass, and related functional readouts as well as of tumor mass and staging [9,76].

In summary, preliminary evidence is presently provided for MAO-A as a potential therapeutic target in cardiac cachexia, since inhibition of MAO-A as a source of H_2_O_2_ by HH reverses high left myocardial expression of atrogin-1 and of the pro-cachectic cytokines IL-1β and TNF as well as SOCS3 and COX2 on the RNA level. The functional significance of such anti-atrophic/-inflammatory HH effects remained open at present. The novel finding of PDAC-related myocardial capillary rarefication, which did not improve with HH treatment, warrants timely clarification regarding its mechanism and functional relevance.

## Figures and Tables

**Table 1:** QuantiTect primer assays used for qPCR.

Primer	Symbol	Amplicon Length (bp)	Cat. Nr.
Actin Beta B cell leukemia/lymphoma 2	ACTB BCL2	77 104	QT01136772 QT02392292
Caspase 3	Casp3	150	QT01164779
CD68 antigen	CD68	67	QT00254051
Atrogin-1 (MAFbx, FBX032))	Atrogin-1	103	QT00158543
Interleukin-1beta	IL-1β	150	QT01048355
Kinase insert domain protein receptor	KDR	95	QT02519972
Monoamine oxidase A	Mao-A	81	QT00109326
Monoamine oxidase B	Mao-B	93	QT00145124
Notch gene homolog 1 (Drosophila)	Notch1	102	QT00156982
Notch gene homolog 3	Notch3	104	QT01051729
Prostaglandin endoperoxide synthase 2	Ptgs2	95	QT00165347
Ribosomal protein lateral stalk subunit P0 Suppressor of cytokine signaling 3	RPLP0 SOCS3	22 90	QT00075012 QT02488990
Tumor necrosis factor alpha	TNF-α	112	QT00104006
Muscle RING-finger protein-1 (TRIM63)	MuRF1	116	QT00291991
Vascular endothelial growth factor A	VEGFA	117	QT00160769

All Primers were purchased from Qiagen N.V., Venlo, the Netherlands.

**Table 2:** Sample size, body weight, and age of wildtype (WT) and PDAC-bearing (CA) mice with or without harmine hydrochloride (HH) treatment.

	WT	WT-HH	CA	CA-HH	ANOVA		
					CA	HH	Interact.
N (m/f)	12 (5/7)	11 (8/3)	10 (6/4)	9 (5/4)	42	42	42
body weight (g)	27.08 ± 1.61	27.17 ± 1.36	27.46 ± 1.09	24.02 ± 0.91 ^#^	*p* = 0.301	*p* = 0.220	*p* = 0.197
age (weeks)	16.58 ± 0.31	18.82 ± 0.70 ^#^	23.20 ± 0.98 ***	19.11 ± 0.48 ^##^	*p* = 0.000	*p* = 0.165	*p* = 0.000

Data are presented as mean ± SEM. Two-factorial ANOVA detected no significant impact of factor CA or factor HH on body weight, which, expectedly, was significantly affected by sex as reported in detail [9,38]. CA mice were significantly ca. 7 and 4 weeks older than WT and CA-HH, respectively, while WT-HH mice were ca. 2 weeks older than WT mice. *** *p* < 0.001 CA vs. WT or CA-HH vs. WT-HH; # *p* < 0.05, ## *p* < 0.01, WT-HH vs. WT or CA-HH vs. CA.

**Table 3:** Expression of pro-atrophic, -inflammatory, -angiogenic, and -apoptotic transcripts in left myocardium of WT-HH, CA, and CA-HH mice relative to the group of untreated WT mice.

	WT	WT-HH	CA	CA-HH
N	8	11	8	9
Monoamino oxidase A (MAO-A)	1.00	0.88	0.72	0.69
Monoamino oxidase B (MAO-B)	1.00	0.91	0.52	0.77
Muscle atrophy F-box (MAFbx)	1.00	1.09	0.84	0.99
Muscle ring finger protein 1 (MURF-1)	1.00	1.30	1.07	1.47
Interleukine-1β (IL-1β)	1.00	0.48 ^#^	2.47 *	1.37
Tumor necrosis factor (TNF)	1.00	0.54	1.87 *	0.35 ^#^
Cyclooxygenase-2 (COX-2)	1.00	1.00	1.53 *	0.41 ^#^
Cluster of differentiation 68 (CD68)	1.00	1.27	1.26	0.72
Suppressor of cytokine signaling 3 (SOCS-3)	1.00	0.48 ^#^	1.64 *	0.39 ^#^
Vascular endothelial growth factor A (VEGF-A)	1.00	1.1	1.02	1.05
Kinase insert domain protein receptor (KDR)	1.00	1.15	0.46 *	0.57
Notch gene homolog 1 (Notch 1)	1.00	0.59	0.68	0.34
Notch gene homolog 3 (Notch 3)	1.00	0.89	0.64	0.38
Caspase-3 (Casp3)	1.00	0.56	1.02	0.49 ^#^
B cell leukemia/lymphoma 2 (Bcl-2)	1.00	1.15	0.97	0.62

The qRT-PCR was conducted in pooled samples per group. A <0.5-fold decrease or >1.5-fold increase was considered relevant and marked by * comparing CA with WT or CA-HH with WT-HH or by # comparing WT-HH with WT or CA-HH with CA.

## Data Availability

The data sets used and/or analyzed during the current study are available from the corresponding author on reasonable request.
